# Epidemiology of Hospitalized Traumatic Pelvic Fractures and Their Combined Injuries in Taiwan: 2000–2011 National Health Insurance Data Surveillance

**DOI:** 10.1155/2014/878601

**Published:** 2014-04-01

**Authors:** Nan-Ping Yang, Chien-Lung Chan, Dachen Chu, Yu-Zhen Lin, Kai-Biao Lin, Ching-Shao Yu, I-Liang Yu, Nien-Tzu Chang, Yi-Hui Lee

**Affiliations:** ^1^Department of Orthopedic Surgery, Taoyuan General Hospital, Ministry of Health & Welfare, Taoyuan 33004, Taiwan; ^2^Institute of Public Health, National Yang-Ming University, Taipei 11221, Taiwan; ^3^Department of Information Management, Yuan Ze University, Taoyuan 32003, Taiwan; ^4^Department of Neurologic Surgery, Taipei City Hospital, Taipei 10341, Taiwan; ^5^Department of Computer Science and Technology, Xiamen University of Technology, Xiamen 361024, China; ^6^Department of Orthopedic Surgery, National Taiwan University Hospital, Taipei 10048, Taiwan; ^7^Department of Nursing, College of Medicine, National Taiwan University, Taipei 10617, Taiwan; ^8^Department of Nursing, School of Nursing, Chang Gang University, Taoyuan 33302, Taiwan

## Abstract

*Background*. From the viewpoint of prehospital emergency medicine, a greater proportion of pelvic fractures not of a life-threatening status but combined with other injuries need more comprehensive recognition. *Methods*. A 12-year nationwide health database of inpatients was reviewed. All cases diagnosed as pelvic fractures were enrolled. The associated injuries classified into 20 categories were further analyzed. *Results*. During 2000–2011, the hospitalized incidence of pelvic fractures in Taiwan ranged from 17.17 to 19.42 per 100,000, and an increasing trend with age was observed. The mean case-fatality rate was 1.6% for females and 2.1% for males; male patients with pelvic fractures had a significantly higher risk of death than female patients after adjusting for other covariates. 74.2% of these cases were combined with other injuries. The most common associated injuries in an identified body region were other orthopedic fractures of the lower limbs (21.50%), spine/trunk (20.97%), or upper limbs (18.18%), followed by significant head injuries (17.59%), intra-abdominal injuries (11.00%), and thoracic injuries (7.20%). *Conclusion*. The incidence of hospitalized pelvic fractures in Taiwan was low and the case-fatality rate was lower than those of other countries. Concurrently, coexistence of major combined injuries with pelvic fractures was easily treated at medical centers.

## 1. Introduction

Pelvic or acetabular fractures are rare injuries (3–8%) [1, 2] as compared with fractures in other body regions. They are accompanied by a high mortality (4–28%) [1, 3–6]. Most deaths in patients with pelvic fractures are not caused by the pelvic fracture itself but are linked to associated injuries [1, 3]. Fatal pelvic injury patients die at a median of 2 days after the trauma [5]. There are significant similarities between pediatric and adult patients with pelvic injuries; the incidences of associated abdominal injuries and the mortality rate of children do not differ from those of adults [1, 7]. A meta-analysis consisting of twelve studies with a total of 5,454 pelvic fracture patients concluded that in stable and alert trauma patients a thorough clinical examination will detect pelvic fractures with nearly 100% sensitivity, thus rendering initial radiography unnecessary in this group of patients [8].

From the viewpoint of prehospital emergency medical management for injured people, identification of pelvic fractures in those with stable or unstable vital signs is critical. Furthermore, evaluation of possible associated injuries is still important even though the greater proportion of pelvic fractures are not of a life-threatening status. Comprehensive epidemiologic surveillance of pelvic fractures with other combined injuries could provide sufficient information to prehospital or inhospital medical staff to improve emergency management and enable policymakers to consider alternative resources and training programs.

In Taiwan, an epidemiological survey using the nationwide randomly sampled database showed that 26.4% of all emergency department (ED) visitors utilized ED services due to injury or poisoning [9]. An emergency-critical hierarchical system (3 levels) was established in Taiwan in 2009. Up to April, 2011, there were 23 highest-level hospitals and 35 midlevel hospitals accredited in Taiwan [10]. Using evidence based on Taiwan's nationwide registered health data, the purposes of the present study were to investigate (1) the incidence and mortality of hospitalized traumatic pelvic fractures in Taiwan, (2) the distributions of other injuries combined with these traumatic pelvic fractures, and (3) factors influencing the pattern of major combined injuries, inhospital mortality and those treated at medical centers among these hospitalized pelvic fractures.

## 2. Methods

### 2.1. Data Resources

From 1995 to 2011, 23.199 million citizens were enrolled in the single-payer National Health Insurance (NHI) program, reaching 99.88% of the total population of Taiwan. This Taiwan universal national health insurance, financed jointly by payroll taxes, subsidies, and individual premiums, has consistently received a 70 percent public satisfaction rate [11]. The nationwide data analyzed in this research were obtained from the National Health Insurance Research Database (NHIRD), which is maintained by the Bureau of National Health Insurance (BNHI) and the National Health Research Institute (NHRI) for research purposes. The academic databank of the NHIRD includes various subdatasets, for example, inpatient expenditures by admissions (DD), details of inpatient orders (DO), ambulatory care expenditures by visits (CD), and details of ambulatory care orders (OO). In this study, the DD dataset was used for further analysis.

### 2.2. Data Protection and Permission

The NHIRD cannot be used to identify patients' personal information; hence, all datasets have already undergone a scrambling procedure before being sent to the NHRI for personal information protection. Essentially, it is impossible to restore the original data when using this database. Moreover, researchers who use the NHIRD are required to declare that they have no intention to obtain information that could potentially violate the privacy of patients or care providers. This study was approved by the Institutional Review Board (IRB) of Taoyuan General Hospital, which has been certified by the Ministry of Health & Welfare, Taiwan (IRB Approval Number: TYGH101049), and the protocol was evaluated by the NHRI, who gave their agreement to this planned analysis of the NHIRD (Agreement Number: NHIRD-101-566).

### 2.3. Data Selection and Definition

In this study, we focused on the population hospitalized due to pelvic fractures, with any other combined injuries or without, data of which were obtained from the nationwide inpatient expenditures by admissions (DD) dataset of the NHIRD between January 1, 2000, and December 31, 2011. All patients meeting the criteria of pelvic fracture with an International Classification of Diseases, 9th Revision, Clinical Modification (ICD9-CM) diagnosis code recorded as 808.X (i.e., 808.0~808.9) were enrolled.

In order to investigate the contribution of categorical diagnoses of associated injuries, 20 groups of ICD9-CM diagnosis codes were evaluated [12]. Thus, combined injuries were defined as (Table 1) fracture of the skull, intracranial injury (800–804, 850–854 series), fracture of the spine and trunk (805–809 series), fracture of upper limbs (810–819 series), fracture of lower limbs (820–829 series), dislocation (830–839 series), sprains and strains of joints and adjacent muscles (840–848 series), internal injury of the chest (860–862 series), internal injury of the abdomen and pelvis (863–869 series), open wound of the head, neck, and trunk (870–879 series), open wound of upper limbs (880–887 series), open wound of lower limbs (890–897 series), injury to blood vessels (900–904 series), late effects of injuries and other external causes (905–909 series), superficial injury (910–919 series), contusion with an intact skin surface (920–924 series), crush injuries (925–929 series), effects of a foreign body entering through an orifice (930–939 series), burns (940–949 series), injury to nerves and the spinal cord (950–957 series), and certain traumatic complications and unspecified injuries (958-959 series).

The pelvic fracture series (ICD9-CM coded as 808.X) was divided into 3 subtypes: (a) acetabulum fracture (coded as 808.0, 808.1) or multiple injuries with disruption of the pelvic ring (coded as 808.43, 808.53); (b) fracture of the pubis (coded as 808.2, 808.3), fracture of the ilium (coded as 808.41, 808.51), or fracture of the ischium (coded as 808.42, 808.52); and (c) others (coded as 808.49, 808.59, 808.8, and 808.9). In order to calculate the operative rate of these pelvic fractures, the ICD9-CM treatment codes were also evaluated.

To evaluate the case-fatality rate of hospitalized pelvic fractures in Taiwan, the mortality of the studied cases was defined as a discharge status coded as died. To evaluate the socioeconomic effect, the enrolled subjects were divided into normal population and low-income population, who must meet the criteria of Taiwan's Social Assistance Act, and were registered in Taiwan's NHI databank.

### 2.4. Statistics

In the analysis in this study, descriptive statistics comparing baseline characteristics were represented by the numbers of cases, percentages, and incidence with the 95% Confidence Interval (CI). Means with Standard Deviation (SD) and Analysis of Variance (ANOVA) were used to describe and compare continuous variables among different subgroups. The significance was set at *P* = 0.05. To evaluate the risk factors of major combined injuries, inhospital mortality and those treated in medical centers among these hospitalized pelvic fractures, multiple logistic regression method was used and Adjusted Odds Ratio (AOR) was calculated. Multilevel analysis (or the hierarchical linear modeling method, HLM method) was used as an analytical strategy, allowing examination of group-level and individual-level factors [13]. The hypothesis and formula of HLM analysis in the present study were shown below as follows.

Level 1 HLM Model

(1) Yij=β0+β1∗(Age)+β2∗(Group-1)+β3∗(Group-2)+β4∗(Group-3)+β5∗(Group-4)+β6∗(Group-7)+β7∗(Group-8)+β8∗(Group-others)+β9∗(Gender)+γ.

Level 2 HLM Model

(2) β0=γ00+γ01∗(hospital-regional)+γ02∗(hospital-medical-center)+μ0.

All statistical analyses were performed using the Statistical Package for Social Sciences for Windows (SPSS for Windows 18.0).

## 3. Results

The hospitalized incidence of pelvic fractures in Taiwan ranged from 17.17 to 19.42 per 100,000 during 2000–2011. Females had a higher incidence than males, and the elderly (aged 65 years or more) were noted to have a significantly increased incidence (Table 2). In total, there were 49,300 incident cases during the 12-year study period enrolled in the present study.

**Figure 1:**
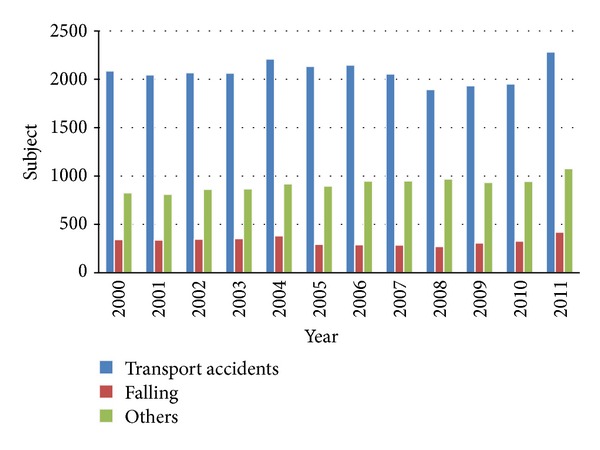
Annual causes of traumatic pelvic fractures admitted for further medical care during 2000–2011 in Taiwan. In summary, 62% of the recorded cases resulted from transport accidents and 10% from falling accidents.

**Figure 2:**
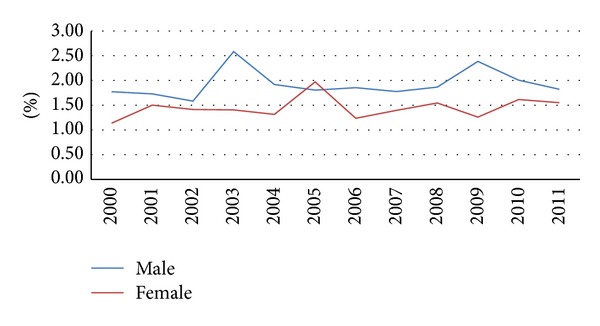
Annual mortality rate of hospitalized pelvic fractures by gender during 2000–2011 in Taiwan.

Figure 1 shows the distribution of causes of traumatic pelvic fractures in the enrolled inpatients. In summary, 62% of the recorded cases resulted from transport accidents and 10% from falling accidents.  Figure 2 shows a dynamic trend of the case-fatality rate during the 12-year period but there is an obvious difference between genders. The mean case-fatality rate of the male patients was 2.1% (ranged from 1.6 to 2.6%), and the mean case-fatality rate of the female patients was 1.6% (ranged from 1.1 to 2.0%).

There were 36,594 cases combined with at least one of the 20 categorical injury groups listed in Table 1; on the other hand, only 25.8% (12,706 cases) of these pelvic fractures were of an isolated pelvic fracture pattern. Among the 20 categories of combined injuries with pelvic fractures, the most common injuries were fractures of lower limbs (cumulative incidence (ci, i.e., risk) and 95% CI: 21.50%, 21.13–21.86%), fractures of the spine/trunk (20.97%, 20.62–21.33%), and fractures of upper limbs (18.18%, 17.84–18.52%). For other specific body regions, the common injuries were fracture of the skull or intracranial injury (17.59%, 17.26–17.93%), internal injury of the abdomen and pelvis (11.00%, 10.72–11.27%), and internal injury of the chest (7.20%, 6.98–7.43%). Focusing on the above six categories of combined injuries (groups 1, 2, 3, 4, 7, and 8), the male patients had a higher incidence of major associated injuries with pelvic fractures than the females, especially chest internal injury (Relative Risk (RR) = 1.76) and abdominal/pelvic internal injury (RR = 1.93) (Table 3).

**Figure 3:**
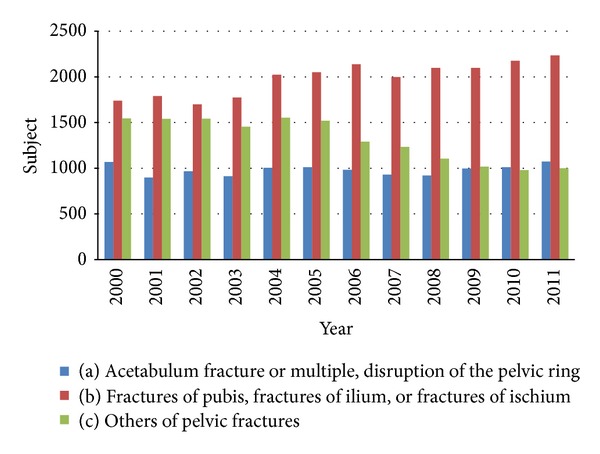
Annual trends of various pelvic fracture patterns during 2000–2011. (The pelvic fractures were classified as (a) acetabulum fracture or multiple injuries with disruption of the pelvic ring; (b) fractures of the pubis, ilium, or ischium; or (c) other pelvic fractures.)

The distributions of the three pelvic fracture patterns were calculated as shown in Figure 3. The most common pelvic fracture pattern was fractures of the pubis, ilium, or ischium. Excluding unspecified fractured locations (coded as others of pelvic fractures), Table 4 compares the medical utilization between more complex types of pelvic fracture (acetabular fracture or multiple injuries with disruption of the pelvic ring) and simple types of pelvic fracture (fractures of the pubis, ilium, or ischium), revealing that a higher operation rate for pelvic fracture (44% versus 22%), a longer length of stay in hospital (average 17.86 days versus 12.95 days), and a greater medical expenditure (average US$4120.86 versus US$2678.09) were noted in the complex pelvic fracture subgroup as compared with the simple pelvic fracture subgroup. Between the complex or simple pelvic fracture patterns, different incidences of the six major associated injuries and their effects on hospitalization days and direct medical cost were also observed, as shown in Table 4.

Among all pelvic fracture cases, the males, the younger (aged 17 years or less), and the 50 years or more groups had less opportunity to suffer any one of the above six major combined injuries. Various pelvic fractures combined with various major associated injuries were also analyzed; an extraordinary dynamic effect of age was found and low-income population was only found to be negatively associated with fractures of the upper limbs (Table 5).

Furthermore, the percentages of operative treatments for pelvic fractures were significantly different among the three levels of hospital (26% versus 17% versus 12% in medical centers, regional hospitals, and district hospitals, respectively; *P* < 0.001). Similarly, the percentages of operative treatments for main combined injuries (any one of injury groups (1), (2), (3), (4), (7), and (8)) were significantly different among the three levels of hospital (70% versus 59% versus 43% in medical centers, regional hospitals, and district hospitals, respectively; *P* < 0.001).

To investigate whether the more severely injured patients were treated at medical centers, a logistic regression model was performed and the hospital level (medical centers versus regional/district hospitals) was used as the dependent variable. As shown in Table 6, model-a, the male patients and younger ages were positively associated with those treated at medical centers; coexistence of other major combined injuries was also easily treated at medical centers (AOR ranged from 1.23 to 2.72; *P* < 0.000). Furthermore, a multilevel analysis was used to evaluate the individual effects (i.e., gender, age, and presence of additional injuries) and the group effect (types of hospitals) on the inhospital deaths (Table 6, model-b); the analysis revealed that male patients with pelvic fractures had a higher risk of death than female patients (AOR = 1.003; *P* < 0.05) after adjusting for other covariates and those treated at medical centers get a higher mortality (AOR = 1.01; *P* < 0.01).

## 4. Discussion

Trauma accounts for approximately 1 in 10 deaths worldwide. The presence of a pelvic fracture increases the mortality risk [14]. In contrast to an overall decline in trauma mortality, complex pelvic ring injuries remain associated with a significant risk of death [15]. In Taiwan, about 10% of annual inpatients were road traffic accident related [12] that indicated the high standing of prehospital medical care for these road injured populations in Taiwan. Some studies have highlighted the importance of effective control of haemodynamic instability to reduce the risk of mortality [4, 16, 17]. The key elements in managing patients with pelvic fractures are swift and adequate resuscitation, reversal of shock and acidosis, and rapid control of hemorrhage to facilitate survival of these patients [16]. From the viewpoint of prehospital emergency medicine, a greater proportion of pelvic fractures not of a life-threatening status, but combined with other injures need more attention and comprehensive recognition. A study that evaluated 2,176 blunt trauma patients showed that 4.5% were diagnosed with a pelvic fracture; among these cases, there were seven missed injuries upon clinical examination (a sensitivity of 93%). This study concluded that clinical examination of the pelvis can reliably rule out significant pelvic fracture in awake and alert blunt trauma patients [18].

A previous study that reviewed 236 pelvic fracture patients showed that 64.4% were injured in motor vehicle accidents, and the average hospital stay was 16.8 days [19]. Another study including 220 men and 128 women with pelvic fractures revealed an average hospital stay of 16.5 days [20]. Similar results were found in the present study: in Taiwan, 62% of hospitalized pelvic fractures were caused by transport accidents, and the mean LOS of these patients was 17.9 days and 13.0 days for a complex and a simple fracture pattern, respectively.

A case series of 348 patients admitted due to pelvic fractures revealed that only 32 patients (9%) had an isolated pelvic fracture [20]. In the present study in Taiwan, as high as 25.8% of hospitalized pelvic fractures were isolated pelvic fractures. Of 1,545 registered pelvic fracture cases, the incidence of abdominal injuries was 16.5%, and, in severe pelvic fractures, the incidence of associated intra-abdominal injuries was 30.7% [3]. A study that included 126 patients with severe pelvic trauma (AO classification type B or C) revealed that the most common extrapelvic lesions were thoracic injuries in 56.4% and severe head injuries (GCS < 8) in 33.3% [6]. In the present study, associated intra-abdominal injuries, thoracic injuries, and significant head injuries were found to be present in 11.0%, 7.2%, and 17.6% of all pelvic fracture cases in Taiwan, respectively. However, greater incidences of other orthopedic fractures (including lower limb, spine/trunk, and upper limb) were noted, at 21.5%, 21.0%, and 18.2% of all pelvic inpatients in Taiwan, respectively. This particular situation has not been reported in the literature and is worthy of further study and policy consideration.

Regression analysis was performed using data of 63,033 patients to assess the odds ratio for mortality associated with pelvic fracture and revealed that hemodynamic shock, severe head injury, and an age of sixty years or more all had an odds ratio for mortality greater than that associated with pelvic fracture [21]. In the present study, a lower case-fatality rate (mean of 1.6% in females and 2.1% in males) was noted, and the emphasis of multiple regression analysis was on cases associated with other injuries, which showed that the male gender and an age of fifty years or more had a lower odds ratio for one of the major combined injuries. In Taiwan, socioeconomic status has been found to have effects on the performance of different invasive treatment methods for hospitalized peripheral arterial disease [22], and a low socioeconomic level population was also noted to have more opportunities to suffer a lower-limb fracture or a spine/trunk fracture among inpatients admitted due to traffic accidents [12]. In the present study, the low-income population was only found to be significantly negatively associated with upper limb fractures when the patients suffered a pelvic fracture.

The NHIB has established a uniform system to control the quality of medical services and coding. If the medical services provided to beneficiaries by the contracted medical care institution are determined by the Professional Peer Review Committee to be incompatible with the provisions of the NHI Act, the expenses thereof are borne by the contracted medical care institution themselves. Otherwise, the Disputes Settlement Board, established under the NHI scheme, settles disputes arising in cases approved by the insurer and raised by the insured, group insurance applicants, or contracted medical care institutions. Based on the above, the quality of data acquisition of the present study would be reliable. However, about 1 third of these pelvic fractures were not accurately classified that made a drawback of the present study.

## 5. Conclusion

The incidence of admission for pelvic fractures was low, and an increasing trend with age was noted in Taiwan. In general, the case-fatality rate of Taiwanese pelvic fractures was lower than those of other countries, and three-quarters of cases were combined with other injuries. The most common associated injuries in an identified body region were other orthopedic fractures of lower limbs, the spine/trunk, or upper limbs, followed by significant head injuries, intra-abdominal injuries, and thoracic injuries. Among the hospitalized pelvic fracture cases, the male patients and younger ages were positively associated with those treated at medical centers, and coexistence of major combined injuries was also easily treated at medical centers.

## Figures and Tables

**Table 1:** Definitions of 20 categories of injuries combined with pelvic fractures*.

Group	ICD9-CM codes	Description
1	800–804 850–854	Fracture of skull (intracranial injury)
2	805–809**	Fracture of spine and trunk
3	810–819	Fracture of upper limb
4	820–829	Fracture of lower limb
5	830–839	Dislocation
6	840–848	Sprains and strains of joints and adjacent muscles
7	860–862	Internal injury of chest
8	863–869	Internal injury of abdomen and pelvis
9	870–879	Open wound of head, neck, and trunk
10	880–887	Open wound of upper limb
11	890–897	Open wound of lower limb
12	900–904	Injury to blood vessels
13	905–909	Late effects of injuries and other external causes
14	910–919	Superficial injury
15	920–924	Contusion with intact skin surface
16	925–929	Crush injuries
17	930–939	Effects of foreign body entering through orifice
18	940–949	Burns
19	950–957	Injury to nerves and spinal cord
20	958-959	Certain traumatic complications and unspecified injuries

*Pelvic fracture series (ICD9-CM coded as 808.X).

**Group 2 associated injuries excluded pelvic fracture series.

**Table 2:** Basic characteristics of cases hospitalized for pelvic fractures in Taiwan, 2000–2011.

Year	Total cases^a^	Total residents^b^	Crude incidence^c∗^	Male	Female	<18 years	18–29.9 years	30–49.9 years	50–64.9 years	65–74.9 years	75 years or more
				Incidence	Incidence	Incidence	Incidence	Incidence	Incidence	Incidence	Incidence
2000	4128	22,276,672	**18.53**	17.33	19.39	4.38	20.85	17.31	25.46	37.53	74.75
2001	4030	22,405,568	**17.99**	16.17	19.44	4.33	20.44	16.24	24.75	37.28	67.08
2002	4001	22,520,776	**17.77**	15.95	19.22	4.13	19.99	15.86	23.52	36.69	67.61
2003	3928	22,604,550	**17.38**	15.81	18.59	3.79	19.67	15.01	23.17	33.22	69.34
2004	4406	22,689,122	**19.42**	17.62	21.13	4.04	22.25	16.71	24.97	40.39	70.51
2005	4384	22,770,383	**19.25**	17.73	20.82	4.02	21.55	16.48	24.92	36.95	71.13
2006	4259	22,876,527	**18.62**	17.21	20.06	4.68	19.22	15.46	23.39	40.11	66.66
2007	4022	22,958,360	**17.52**	15.52	19.56	4.20	18.73	13.33	21.41	36.26	70.40
2008	3956	23,037,031	**17.17**	15.67	18.70	3.70	18.27	12.89	21.12	36.44	66.59
2009	3985	23,119,772	**17.24**	15.13	19.37	3.33	18.85	12.22	21.88	36.79	64.72
2010	4025	23,162,123	**17.38**	15.43	19.35	3.72	17.47	13.75	20.36	33.92	66.32
2011	4176	23,224,912	**17.98**	16.50	19.47	3.62	19.07	13.38	20.07	37.07	69.68

c = a/b; *1/100,000.

**Table 3:** Descriptions of the top 10 major injuries associated with hospitalized pelvic fracture cases in Taiwan.

Categorical diagnoses of combined injuries (grouping)	Summed cases in 2000–2011			Cumulative incidence in general	Cumulative incidence in males	Cumulative incidence in females
	Number	Male (%)	Female (%)	Percentage (95% CI)	Percentage (95% CI)	Percentage (95% CI)
(4) Fracture of lower limb	10597	53.89	46.11	**21.50 (21.13–21.86)**	25.23 (24.66–25.79)	18.49 (18.02–18.96)
(2) Fracture of spine and trunk	10340	49.61	50.39	**20.97 (20.62–21.33)**	22.59 (22.04–23.13)	19.66 (19.18–20.14)
(3) Fracture of upper limb	8961	49.63	50.37	**18.18 (17.84–18.52)**	19.57 (19.05–20.08)	17.02 (16.56–17.47)
(15) Contusion with intact skin surface	8889	43.50	56.50	**18.03 (17.69–18.37)**	16.98 (16.50–17.47)	18.90 (18.43–19.37)
(1) Fracture of skull (intracranial injury)	8674	46.72	53.28	**17.59 (17.26–17.93)**	17.81 (17.32–18.31)	17.41 (16.95–17.87)
(9) Open wound of head, neck, and trunk	6243	55.32	44.68	**12.66 (12.37–12.96)**	15.17 (14.70–15.63)	10.50 (10.13–10.86)
(14) Superficial injury	5789	42.67	57.33	**11.74 (11.46–12.03)**	10.85 (10.45–11.26)	12.50 (12.10–12.90)
(8) Internal injury of abdomen and pelvis	5421	62.36	37.64	**11.00 (10.72–11.27)**	14.82 (14.36–15.29)	7.67 (7.35–7.99)
(20) Certain traumatic complications and unspecified injuries	3830	48.82	51.18	**7.77 (7.53–8.01)**	8.23 (7.87–8.59)	7.39 (7.08–7.71)
(7) Internal injury of chest	3551	60.12	39.88	**7.20 (6.98–7.43)**	9.40 (9.02–9.78)	5.34 (5.07–5.61)

CI: confidence interval.

**Table 4:** Medical utilization of hospitalized pelvic fracture cases with major combined injuries in Taiwan.

Complex or simple type of pelvic injury	Group of combined injury	Summed cases 2000–2011 (%)	Operation rate for the pelvic injury (%)	Length of stay (days)	Medical cost (US$)
				Mean (SD)	Mean (SD)
Acetabular fracture or multiple injuries with disruption of the pelvic ring (a)	Combined major injuries or not		**44%**	**17.86 (14.31)**	**4120.86 (4902.90)**
	Combined with injury group-(1)	**18.12%**	39%	16.28 (14.10)	3726.21 (4673.87)
	Combined with injury group-(2)	**15.58%**	34%	17.10 (13.98)	3510.86 (4457.71)
	Combined with injury group-(3)	**16.11%**	45%	16.91 (12.88)	3605.73 (3918.62)
	Combined with injury group-(4)	**28.22%**	53%	18.62 (14.29)	4389.91 (4961.58)
	Combined with injury group-(7)	**6.40%**	42%	19.88 (14.85)	5291.04 (5946.72)
	Combined with injury group-(8)	**8.72%**	42%	20.30 (16.70)	5252.62 (6166.91)
ANOVA test				*P* = 0.000	*P* = 0.000

Pubic, ilium, and ischium (b)	Combined major injuries or not		**22%**	**12.95 (11.40)**	**2678.09 (3568.46)**
	Combined with injury group-(1)	**19.62%**	8%	11.16 (10.70)	2217.28 (3276.76)
	Combined with injury group-(2)	**21.75%**	3%	12.90 (11.34)	2259.66 (3209.70)
	Combined with injury group-(3)	**17.37%**	32%	11.59 (9.91)	2216.78 (2852.88)
	Combined with injury group-(4)	**16.37%**	65%	14.68 (12.34)	3455.27 (3972.13)
	Combined with injury group-(7)	**5.70%**	18%	16.19 (12.48)	3998.03 (4536.55)
	Combined with injury group-(8)	**8.81%**	16%	14.43 (12.16)	3348.78 (4185.53)
ANOVA test				*P* = 0.000	*P* = 0.000

**Table 5:** Multiple logistic regression models of hospitalized pelvic fractures with the six major combined injuries in Taiwan.

	Pelvic fractures with any of the 6 major combined injuries	Pelvic fractures with injury group-(1)	Pelvic fractures with injury group-(2)	Pelvic fractures with injury group-(3)	Pelvic fractures with injury group-(4)	Pelvic fractures with injury group-(7)	Pelvic fractures with injury group-(8)
	AOR (95% CI)	AOR (95% CI)	AOR (95% CI)	AOR (95% CI)	AOR (95% CI)	AOR (95% CI)	AOR (95% CI)
Gender							
Female	**1**	**1**	**1**	**1**	**1**	**1**	**1**
Male	**0.759 (0.731–0.789)**	**1.101 (1.051–1.153)**	**0.866 (0.826–0.907)**	**0.884 (0.842–0.928)**	**0.733 (0.700–0.768)**	**0.596 (0.553–0.642)**	**0.594 (0.558–0.633)**
Age stratum							
≤17 y/o	**0.559 (0.511–0.611)**	**0.863 (0.776–0.960)**	**0.384 (0.325–0.454)**	**0.688 (0.602–0.787)**	**0.688 (0.615–0.770)**	**0.624 (0.514–0.758)**	1.018 (0.903–1.149)
18–29 y/o	**1**	**1**	**1**	**1**	**1**	**1**	**1**
30–49 y/o	0.983 (0.931–1.037)	**0.803 (0.754–0.854)**	**1.581 (1.477–1.692)**	**1.157 (1.080–1.240)**	0.924 (0.869–0.983)	1.052 (0.954–1.159)	**0.703 (0.651–0.759)**
50–64 y/o	**0.870 (0.821–0.921)**	**0.742 (0.693–0.794)**	**1.637 (1.523–1.759)**	**1.339 (1.245–1.441)**	**0.685 (0.639–0.735)**	1.023 (0.920–1.139)	**0.480 (0.438–0.527)**
65–74 y/o	**0.675 (0.632–0.721)**	**0.662 (0.610–0.718)**	**1.359 (1.249–1.480)**	**1.092 (1.000–1.191)**	**0.612 (0.562–0.667)**	**0.762 (0.665–0.874)**	**0.337 (0.297–0.382)**
≥75 y/o	**0.447 (0.421–0.476)**	**0.410 (0.377–0.446)**	1.035 (0.953–1.125)	**0.705 (0.644–0.771)**	**0.521 (0.481–0.566)**	**0.405 (0.347–0.474)**	**0.200 (0.174–0.231)**
Socioeconomic level							
Normal population	1	1	1	1	1	1	1
Low-income population	0.918 (0.786–1.073)	0.994 (0.817–1.209)	1.016 (0.833–1.239)	**0.791 (0.631–0.991)**	1.011 (0.831–1.230)	1.209 (0.903–1.620)	1.018 (0.783–1.322)

AOR: adjusted odds ratio.

**Table 6:** Multiple logistic regression model^a^ of treated-at-medical centers and multilevel analysis (HLM method)^b^ of inhospital mortality among the hospitalized pelvic fractures in Taiwan.

	Dependent variable = treated-at-medical centers^a^				Dependent variable = inhospital mortality^b^			
	*β* value	*P* value	AOR (95% CI)		*β* value	*P* value	AOR (95% CI)	
Gender (male versus female)*	0.151	0.000	**1.116 (1.116–1.211)**		0.003	0.024	**1.003 (1.000–1.005)**	
Age (years)*	−0.010	0.000			0.000	0.004		
Additional injuries*								
Simple pelvic fractures (fx)			1.0				1.0	
Pelvic fx with injury group-(1)	0.437	0.000	**1.547 (1.365–1.754)**		0.039	0.000	**1.040 (1.027–1.053)**	
Pelvic fx with injury group-(2)	0.203	0.000	**1.225 (1.111–1.351)**		0.005	0.016	**1.005 (1.001–1.009)**	
Pelvic fx with injury group-(3)	0.341	0.000	**1.407 (1.287–1.539)**		0.004	0.038	**1.004 (1.000–1.008)**	
Pelvic fx with injury group-(4)	0.527	0.000	**1.694 (1.569–1.828)**		0.013	0.000	**1.013 (1.009–1.017)**	
Pelvic fx with injury group-(7)	0.999	0.000	**2.715 (2.414–3.054)**		0.062	0.000	**1.064 (1.051–1.077)**	
Pelvic fx with injury group-(8)	0.849	0.000	**2.337 (2.134–2.559)**		0.052	0.000	**1.053 (1.040–1.067)**	
Pelvic fx with other injuries	−0.219	0.000	**0.803 (0.762–0.847)**		0.013	0.000	**1.013 (1.010–1.017)**	
Hospital level**								
District hospital							1.0	
Regional hospital					−0.008	0.000	**0.992 (0.989–0.995)**	
Medical center					0.010	0.007	**1.010 (1.003–1.017)**	

*Individual level.

**Cluster level.
